# Serum metabolomic signatures discriminate early liver inflammation and fibrosis stages in patients with chronic hepatitis B

**DOI:** 10.1038/srep30853

**Published:** 2016-08-08

**Authors:** Haijun Huang, Zeyu Sun, Hongying Pan, Meijuan Chen, Yongxi Tong, Jiajie Zhang, Deying Chen, Xiaoling Su, Lanjuan Li

**Affiliations:** 1 State Key Laboratory for Diagnosis and Treatment of Infectious Diseases, Collaborative Innovation Center for Diagnosis and Treatment of Infectious Diseases, The First Affiliated Hospital, College of Medicine, Zhejiang University, 310003 Hangzhou, People’s Republic of China; 2 Department of Infectious Diseases, Zhejiang Provincial People’s Hospital, 310014 Hangzhou, People’s Republic of China

## Abstract

Chronic HBV (CHB) infected patients with intermediate necroinflammation and fibrosis are recommended to receive antiviral treatment. However, other than liver biopsy, there is a lack of sensitive and specific objective method to determine the necroinflammation and fibrosis stages in CHB patients. This study aims to identify unique serum metabolomic profile associated with histological progression in CHB patients and to develop novel metabolite biomarker panels for early CHB detection and stratification. A comprehensive metabolomic profiling method was established to compare serum samples collected from health donor (n = 67), patients with mild (G < 2 and S < 2, CHB1, n = 52) or intermediate (G ≥ 2 or S ≥ 2, CHB2, n = 36) necroinflammation and fibrosis. Multivariate models were developed to differentiate CHB1 and CHB2 from controls. A set of CHB-associated biomarkers was identified, including lysophosphatidylcholines, phosphatidylcholines, phosphatidylinositol, phosphatidylserine, and bile acid metabolism products. Stratification of CHB1 and CHB2 patients by a simple logistic index, the PIPSindex, based on phosphatidylinositol (PI) and phosphatidylserine (PS), was achieved with an AUC of 0.961, which outperformed all currently available markers. A panel of serum metabolites that differentiate health control, CHB1 and CHB2 patients has been identified. The proposed metabolomic biosignature has the potential to be used as indicator for antiviral treatment for CHB management.

Patients with chronic hepatitis B (CHB) are at high risk of developing hepatic decompensation, liver cirrhosis, hepatocellular carcinoma (HCC) and end-stage liver disease (ESLD)^1^,^2^. Antiviral treatment can suppress HBV replication and rescue acute exacerbations of CHB, and prevent progression of CHB to cirrhosis, HCC and ESLD^3^,^4^,^5^,^6^. However, the reversion from inactive CHB to active states can occurs spontaneously without perceivable symptoms^5^. The current guidelines proposed by American Association for the Study of Liver Diseases (AASLD), the European Association for the Study of the Liver (EASL) and the Asian-Pacific Association for the Study of the Liver (APASL) for antiviral treatment predominantly rely on monitoring alanine aminotransferase (ALT) level^7^,^8^,^9^. Despite being adopted widely, there is still a dearth of studies to evaluate these guidelines for CHB management. Recent doubts have been raised that there might be a sizeable portion of CHB subgroups that will benefit from, but were unfortunately not eligible for antiviral treatment under the current frameworks^9^,^10^,^11^,^12^,^13^,^14^. Our previous data together with reports from others showed that marked necroinflammation (grade G ≥ 2) and fibrosis (stage S ≥ 2) may not trigger ALT increase in many CHB patients, who were not eligible for antiviral treatment^15^,^16^,^17^,^18^. So far, liver biopsy (LB) is the golden standard for assessing liver inflammation and fibrosis. But the invasive nature of this procedure, alongside concerns with sampling error and assessment variability make it unsuitable for CHB evaluation and monitoring in large population^19^,^20^. Therefore, there is need to develop noninvasive biomarkers to accurately assess the early CHB stages. The majority of published serum biomarkers were proposed for detecting significant liver fibrosis in CHB patients^21^, only a few serum biomarkers were proposed to assist in the detection of liver necroinflammation in CHB patients^22^,^23^, but these serum biomarkers all have not been tested to predict the overall histological severity of CHB.

Metabolomics is an established systematic approach to profile metabolites in any given biological samples and leads disease markers generation. Recent metabolomic studies based on ultra-performance liquid chromatography coupled with high-resolution mass spectrometry (UPLC-HRMS) have helped to develop diagnostic or prognostic biomarkers for a variety of liver diseases^24^,^25^,^26^,^27^. We postulate that mild hepatic inflammation and fibrosis at early CHB stages will lead to liver metabolic shifts without extensive cellular damages, and can be reflected by serum metabolomic alterations. In this study, we specifically aimed to develop multivariate models using high coverage UPLC-HRMS metabolomics data to differentiate patients with mild or significant inflammation and fibrosis stages within CHB cohort with ALT level smaller than 2X ULN and healthy controls. Based on this model, we further aimed to develop serum metabolite markers for CHB stage stratification.

## Results

### Study cohort characteristics

Of all 155 subjects (52 CHB1, 36 CHB2 and 67 normal, Table 1), about 2/3 were used for model training, while the remaining 1/3 were used for model validation. Patients in all groups were well matched with respect to age, gender ratio, and there were no remarkable differences in ALB, GLB, Cr, BUN levels. Importantly, CHB1 and CHB2 patients showed comparable HBeAg and HBV DNA level. When compared to healthy controls and CHB1 patients, CHB2 patients had significant higher level of ALT, AST, GGT and AKP, and significant lower level of PLT as expected.

Hepatic biopsies were obtained from all 88 CHB patients. In summary, 18.6% (11/59) of the training set and 24.1% (7/29) of the validation set had significant fibrosis (S2–4), while 38.9% (23/59) of the training set and 34.5% (10/29) of the validation set had significant inflammation (G2–4). Examples of liver biopsy histology from CHB1 and CHB2 patients are shown in Supplementary Figure 1.

### UPLC-HRMS profiling of serum metabolome

All serum samples from both training and validation sets were analyzed using UPLC-HRMS nontargeting profiling method. The typical UPLC-HRMS chromatogram can be found in Supplementary Figure 2a. Instrument performance was constantly monitored by QC sample injections. Given the high stability of the UPLC and MS performance, successful alignment of metabolomic profiles between samples was achieved (Supplementary Figure 2b). The resulting 4636 unique RT-m/z features were subsequently used for multivariate analyses.

### Initial multivariate model based on all detected features

**Figure 1:**
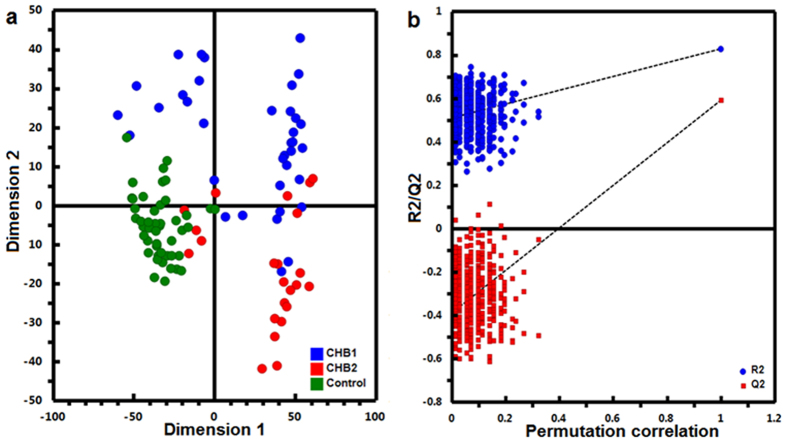
PLS-DA model of the training set. The PLS-DA score plot showed clear separation of 3 groups. (**a**) Verification of the PLS-DA model by a class permutation tests. (**b**) The horizontal axis indicates the correlation between the ‘real’ and the permuted ‘y’ class. The vertical axis represents R2 (goodness-of-fit) and Q2 (goodness-of-prediction) values of each permuted model.

To illustrate CHB-related metabolomic alterations, a supervised PLS-DA model were constructed using the training set (Fig. 1a). Using 7-fold cross-validation (CV), this model achieved 78% goodness-of-fit (R^2^Y) with a goodness-of-prediction (Q^2^) of 62%. Class permutation test also indicated that the model was rigorously built without overfitting (Fig. 1b). However, this full PLS-DA model cannot distinguish all 3 groups completely, albeit an overall separation trend was showed. Considering the complex and dynamic nature of human serum metabolome, this is possible that our data still comprised majorly innegligible individual differences, i.e. disease irrelevant variations, whereas the inflammation and fibrosis at such early stages are unlikely to introduce dominant impact on global metabolite profiles.

### Selection and characterization of potential CHB biomarkers

Potential biomarkers contributed to the discriminative power were selected according to VIP score, which measures the importance of individual variables in the projection used in the PLS-DA model. Extra stringent criteria of VIP > 2 and significant intergroup differences in normalized MS intensity (*t*-test, *P* < 0.05) as well as quality filtering steps described in Supporting Information were taken to narrow down the targets to a final list of 26 metabolite features depicted in Table 2.

**Figure 2:**
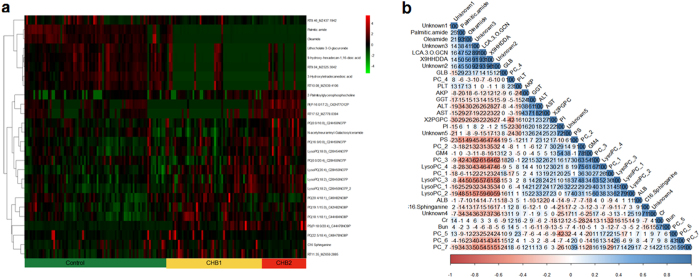
Heatmap (**a**) representation of clustering of 26 discriminating metabolites across the 3 groups of patients (CHB1 and CHB2 in yellow and red, healthy controls in green). Columns represent individual samples and rows refer to distinct metabolites. Shades of red or green represent elevation or decrease, respectively, of a metabolite relative to the median metabolite levels. Correlation matrix (**b**) of 26 discriminating metabolites and 7 clinical serum markers (ALT, AST, GGT, AKP, PLT, GLB, ALB) based on their abundance profiles across all samples. Shades of red or blue represent low-to-high correlation coefficient between markers.

Using tandem MSMS spectra and database matching, a total of 21 compounds, mostly lysophosphatidylcholine, phosphatidylcholine, fatty acid or bile acid metabolites were identified (Table 2). The identity of remaining 5 compounds cannot be revealed at this point, due to lack of record in current databases. The relative intensities of these 26 metabolites across samples were displayed by heatmap (Fig. 2a). There were 21 and 18 metabolites shown significant differences between CHB1 and healthy controls, and between CHB1 and CHB2, respectively. Correlation analysis (Fig. 2b) suggested no significant dependence of metabolite markers on any current serological markers. Interestingly, AST, ALT and GGT were highly correlated in our dataset. In addition, we identified two highly correlated clusters of metabolites: one included palmitic amide, oleamide, lithocholate 3-O-glucuronide and 9-hydroxy-hexadecan-1,16-dioic acid (9HHDDA), while the other one comprised mostly lysophosphatidylcholines and phosphatidylcholines. A negative correlation between these two metabolite groups was observed.

Complementary functional analysis was performed in addition to the identified metabolites using all significantly changed RT-m/z features. Overall, we found several fatty-acid metabolism pathways are highly represented in the 455 significantly changed m/z species selected from the previous PLS-DA model (Supplementary Table 3). These results highly correlated with the CID evidence which indeed identified mainly products from fatty acids metabolism.

### Differentiate CHB groups using simplified OPLS-DA models

**Figure 3:**
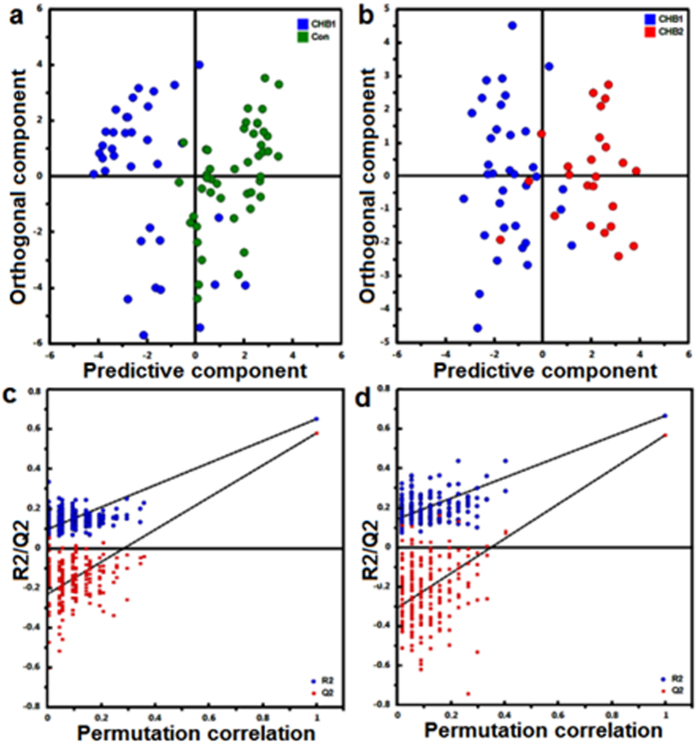
OPLS-DA models, built with short list of 21 CHB1vsCon specific (**a**) and 18 CHB2vsCHB1 specific (**b**) m/z species. Validation of the OPLS-DA models by class permutation tests (**c,d**).

**Figure 4:**
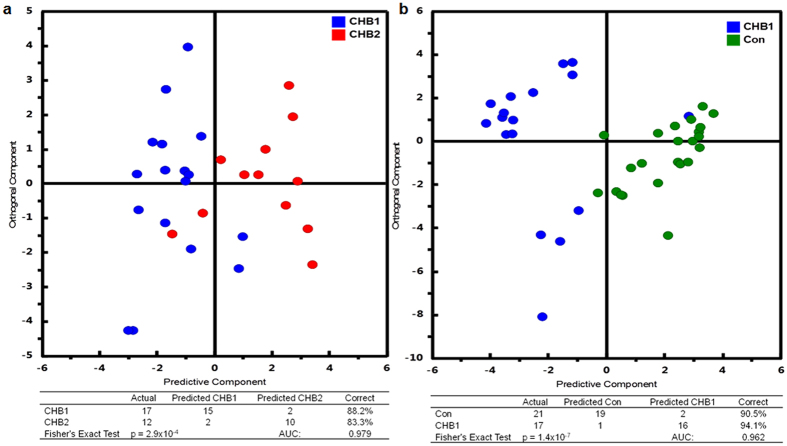
The OPLS-DA predicted class for the CHB1vsCon (**a**) and CHB2vsCHB1 (**b**) validation sets.

Subsets of metabolites shown significant intergroup differences were used to build simplified OPLS-DA models to replace the full PLS-DA model. The 1^st^ OPLS-DA model (R^2^Y = 0.65, Q^2^ = 0.58) was established based on 21 metabolites showing significant difference between CHB1 and controls (Fig. 3a). Using this model, 30 out of 35 (85.7%) CHB1 and 42 out of 46 (91.3%) control samples were correctly classified. Using the 2nd OPLS-DA model (R^2^Y = 0.67, Q^2^ = 0.57) built on 18 metabolites showing significant difference between CHB1 and CHB2, 31 out of 35 (88.6%) CHB1 and 21 out of 24 (87.5%) CHB2 samples were correctly classified (Fig. 3b). Both OPLS-DA model using one predictive and one orthogonal latent variables achieved clear intergroup separation with comparable fitting and predictive power to the original PLS-DA model based on all RT-M/Z features (Supplementary Table 1). In addition to class permutation test that shown the reliability of both OPLS-DA models (Fig. 3c,d), a set of validation samples (i.e. 17 CHB1, 12 CHB2 and 21 controls) was used to prospectively evaluate the predictability of these two OPLS-DA models. The results revealed that 94.1% CHB1 samples and 90.5% controls were correctly predicted using the 1^st^ OPLS-DA model, and 88.2% CHB1 and 83.3% CHB2 samples were correctly predicted using the 2^nd^ OPLS-DA model (Fig. 4a,b).

### Receiver operating characteristic curve analyses

Receiver operating characteristic (ROC) curve analyses were performed for individual markers and possible marker combinations. We focused our analysis on markers with known chemical formula and have an AUC > 0.7: PS, PI, GM4, LysoPC_1 and PC_5 (Supplementary Table 2). PI (AUC = 0.87) displayed a mediocre sensitivity at 75%, but nonetheless has the highest specificity (100%) among all others. On the contrary, PS (AUC = 0.71) provides superior sensitivity (100%), but lacks sensitivity (44.23%). We propose these two biomarkers can be used complementarily. Among conventional serum biomarkers, the AST shown the best combination of both sensitivity (69.44%) and specificity (76.92%) with AUC reached 0.765. Such distinctive diagnostic characteristics of different markers identified in this dataset prompt us to try marker combinations to achieve higher sensitivity and specificity for CHB stratification. Regarding to this, we further constructed a logistic regression model, dubbed PIPSindex (Equation 1), using combinations of the relative MS intensity from 2 metabolites.


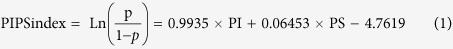


**Figure 5:**
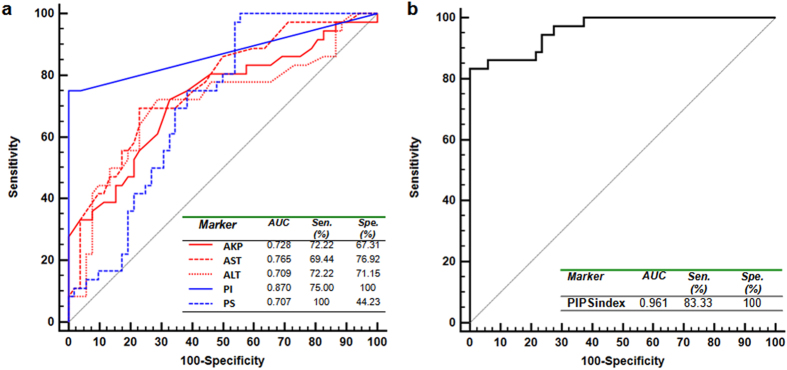
Area under the receiver operating characteristic (ROC) curves, comparing diagnostic performance of PI, PS with serum ALT, AST, AKP (**a**) and the logistic PIPSindex (**b**) to differentiate CHB2 from CHB1. AUC, Area under the curve; Sen, Sensitivity; Spe, Specificity.

We defined odds of dichotomous classification by the probability of being classified as CHB2 (p) divided by the probability of being classified as CHB1 (1-p). The PIPSindex resulted in much balanced sensitivity (83.33%) and specificity (100%) with AUC reached 0.961. In our data, this logistic regression index based on 2 metabolite panel outperformed the current serological marker such as ALT and AST (Fig. 5b, Supplementary Table 2). In addition, LysoPC _1 (AUC = 0.74), PC_5 (AUC = 0.77) and GM4 (AUC = 0.74) also shown comparable diagnostic value as aminotransferases. Yet combinations of these variables did not have significant improvement over sensitivity and specificity.

## Discussion

One of the biggest conundrum with chronic viral hepatitis management is when to start or who will benefit from the antiviral treatment. For many CHB patients, liver histology is not always available, therefore surrogate biomarker, like ALT was used to evidence the need of antiviral treatment. Nevertheless, previous studies suggested about half of CHB patients have significant inflammation (G ≥ 2) or fibrosis (S ≥ 2) shown normal and mildly elevated ALT values (≤2 ULN)^15^,^16^,^17^,^18^. However, the current study suggested even higher percentage of CHB patients with significant inflammation or fibrosis stages (CHB2, 32 out of 36) would failed to be diagnosed by ALT without extra histopathological evidence. Based on these observations, we argue that ALT value alone lacks the sensitivity to determinate the active inflammation in a certain portion of CHB patients. We reasoned that the release of ALT only occurs upon distortion of hepatic membrane permeability which correlates with severe histological damages. In comparison, small molecules can be readily transported or diffuse through cellular boundaries, thus reflecting subtle biochemical alterations in hepatocytes with much higher sensitivity.

The liver functions as the “chemical factory” of the body and contributes significantly to the metabolic content pool in the blood. We therefore proposed that pathological alteration, such as fibrosis and inflammation induced by CHB, can be reflected in the changes of serum metabolic profiles. Profiling different classes of biochemicals simultaneously, i.e. metabolomics, has gain popularity by the significant technological advances in analytical instrumentation and methodologies recently. When coupled with multivariate analyses, metabolomics has emerged as an powerful tool to characterize disease phenotypes, to identify novel biomarkers, and to understand the mechanisms underlying pathological progression. A plethora of metabolomic investigations has been attempted to study liver diseases^25^,^26^,^27^. To the best of our knowledge, this is the first study to investigate the relationship between global serum metabolomic signatures and histologic characteristics in CHB patients. We hope this study can help to lay the foundation for future development of novel, sensitive, and none-invasive circulating diagnostic biomarkers to foresee the overall hepatic histological severity, and hence to guide antiviral therapy for CHB patients.

Quantitative global metabolomic survey in coupled with pathway analysis suggest CHB cause significant shift in fatty acid, vitamin A/E, and amino acid metabolism, which all take place in the liver. In addition, the identified biomarkers also suggested that remarkable changes in the levels of lysophosphatidylcholines, phosphatidylcholines, sphingomyelins and bile acid metabolism products. In particular, we found lysophosphatidylcholines including LysoPC_1 were highly elevated in CHB2 patients suggests extensive cell death, as they have been well documented as toxic metabolite markers as the result of hepatocytes apoptosis^28^,^29^. Phosphatidylserine (PS) also plays important role in cellular apoptosis, and attract macrophages to engulf actions during tissue damage^30^. In addition, conjugated bile acid lithocholate-glucuronide has also been shown cytotoxic and plays important roles in bile acid and very-low-density lipoproteins transportation across hepatocyte membrane^31^,^32^. N-acetylneuraminyl-Galactosylceramide (Sialyl-GalCer, GM4) is a key byproduct of sphingolipid biosynthesis. Although sphingolipid metabolism has long been indicated in liver disease progression^33^,^34^,^35^, yet the specific role of GM4 in hepatitis has not reported, therefore future studies are required to investigate the relationship between GM4 alteration and chronic HBV infection.

One of the key aims of this study is to develop biomarker panels for CHB stratification in early stages. To fulfill this end, OPLS-DA models based on a subset of 18 metabolites was built to specifically discriminate CHB2 from CHB1 patients, with an excellent predictive power with an AUC of 0.979 in the validation sample set. In addition, when compared to healthy controls within the validation sample set, the serum samples from CHB1 patients can be distinguished with an AUC of 0.962 using the OPLS-DA model based on a subset of 21 metabolites. The predictive capability of both models was further tested in the validation datasets with >85% accuracy. These results support the hypothesis that serum metabolomic signatures could be useful to reflect histologic changes in CHB patients.

However, it is understood that total metabolomic profiles cannot be used directly for clinical diagnostic purpose in large-scale, due to uncontrollable variations caused by instruments, workflows and sophisticated data mining process. Therefore, key metabolite biomarkers that contributed most to the overall intergroup metabolomic differences should be selected as surrogate targets, based on which simple and robust diagnostic assays can be developed. To this end, ROC analyses were performed for each metabolite candidate in comparison with current available biomarkers (Supplementary Table 2). Interestingly, the ALT (AUC = 0.709) did not perform the best among these biochemical indicators, while the AST (AUC = 0.765) shown improved diagnostic value for CHB2 (Fig. 5a). In comparison, we found individual metabolic markers only provide compromised diagnostic performance with sensitivity and specificity trade-offs. In our datasets, PIPSindex (AUC = 0.961), comprised of PI and PS, outperformed ALT or AST with much improved sensitivity and specificity as shown by Fig. 5b. In summary, the metabolomics approach described herein has allowed us to stratify CHB patients at early stage with high degree of agreement to the histological results.

Attempting to bridge the gap between professional society guidelines and expert recommendations regarding which CHB patient should be treated and which patient can be monitored, this work aims to unravel unique metabolomic signature and to discover novel serum metabolite constituents associated with CHB development. Combinatory metabolites panel for patient stratification at early CHB stages were developed. Further mechanistic investigations on how these metabolites involved with the CHB progression and histologic changes are clearly warranted. Moreover, further validation using targeted methods such as multiple-reaction monitoring on LC-MSMS platform in larger CHB cohort are needed to evaluate the performance of these markers.

## Materials and Methods

### Clinical samples

Eighty-eight consecutive treatment naive CHB patients were prospectively enrolled in Department of infection disease, Zhejiang provincial people’s hospital from June 2012 to December 2013. Inclusion criteria were age ≥20 years, positive HBsAg for more than 6 months, HBV DNA ≥10^3^ copies/ mL and ALT ≤2 ULN (ULN = 50 U/L); ALT and HBV DNA were monitored monthly for 6 months prior to enrollment to ensure the persistent maintenance of ALT ≤2 ULN and HBV DNA ≥10^3^ copies/mL. The control group included 67 healthy individuals who came to the hospital for medical evaluation. They were confirmed to have normal liver function without any liver diseases. Informed consent was obtained from all patients. The study protocol was carried out in accordance with the guidelines approved by Ethics Committee of the Zhejiang Provincial People’s Hospital and the ethical guidelines of the 1975 Declaration of Helsinki. Exclusion criteria and serum sample collection protocol is detailed in the Supporting Information.

### Biochemical and histopathological analysis

Circulating marker such as alanine aminotranferease (ALT), aspartate aminotransferase (AST), alkaline phosphatase (ALP), gamma-glutamyl transferase (GGT), total bilirubin (Tbil), albumin (ALB), together with serum HBV-DNA and hepatitis antibodies including HBsAg, HBsAb HBeAg, HBeAb, HBcAb, and anti-HCV were measured in the clinical laboratory detailed in Supporting Information.

All enrolled patients received LB were staged by liver necroinflammation activity (G0-G4) and liver fibrosis (S0–S4) using Scheuer’s classification^36^ as detailed in Supporting Information. Patients were divided into two groups with different histological severity levels: CHB1 (mild CHB with G ≤ 1 and S ≤ 1) and CHB2 (severe CHB with G ≥ 2 or S ≥ 2).

### Serum metabolomics analysis

Serum metabolite fingerprinting, data processing was performed on a UPLC-Q-TOF platform using parameters detailed in the Supporting Information.

### Multivariate modeling and statistical analyses

Partial Least Squares Projection to Latent Structures regression with Discriminant Analysis (PLS-DA)^37^ was used to extract relevant intergroup associations in the metabolomics data. Further simplified orthogonal PLS-DA models (OPLS-DA)^38^ based on short lists of markers that differentiate CHB2 from CHB1 and CHB1 from controls were built. Continuous clinical and biochemical data were compared by one-way ANOVA or student’s *t*-test, while categorical data were compared using Chi-square test. Significance was established by *P* < 0.05. The reader is referred to Supporting Information for details.

### Biomarker identification and pathway analysis

Biomarkers candidate for CHB staging were selected based on PLS-DA model by VIP > 2 (Variable Importance in the Project score) and a set of additional criteria. Selected metabolites were identified by comparing their exact mass or MS/MS spectra to public metabolite reservoirs as described in Supporting Information.

An additional strategy was employed to unveil inflammation and fibrosis related metabolic pathway during CHB progression using the mummichog approach^39^ by mapping significantly alterated RT-m/z features to reference human metabolic networks in public domain. The reader is referred to Supporting Information for details.

## Additional Information

**How to cite this article**: Huang, H. *et al.* Serum metabolomic signatures discriminate early liver inflammation and fibrosis stages in patients with chronic hepatitis B. *Sci. Rep.* **6**, 30853; doi: 10.1038/srep30853 (2016).

## Supplementary Material

Supplementary Information

## Figures and Tables

**Table 1:** Clinical characteristics of enrolled patients.

Groups	Training set				Validation set			
	CHB1 (n = 35)	CHB2 (n = 24)	Control (n = 46)	P-Value*	CHB1 (n = 17)	CHB2 (n = 12)	Control (n = 21)	P-Value*
Age (yr)	37.14 ± 9.68	0.42.13 ± 12.33	39.30 ± 9.43	>0.05	31.94 ± 9.22	36.83 ± 10.29	36.81 ± 8.50	>0.05
Gender (M:F)	23:12	15:9	32:14	>0.05†	6:11	8:4	12:9	>0.05†
ALT (IU/L)	34.34 ± 18.41	55.83 ± 42.63	23.96 ± 10.12	**<0.01**	27.65 ± 17.47	48.42 ± 27.48	23.42 ± 9.94	**<0.01**
AST (IU/L)	29.26 ± 10.44	50.13 ± 39.29	23.91 ± 5.27	**<0.01**	26.297 ± 11.98	37.08 ± 14.75	25.81 ± 4.83	**<0.05**
GGT (IU/L)	23.26 ± 14.19	45.63 ± 44.34	23.76 ± 11.81	**<0.01**	18.29 ± 9.31	34.17 ± 31.88	21.14 ± 5.82	**<0.05**
AKP (IU/L)	74.00 ± 13.90	91.25 ± 28.56	66.11 ± 17.78	**<0.01**	62.00 ± 15.46	81.08 ± 20.82	63.38 ± 16.98	**<0.05**
ALB (g/L)	46.36 ± 4.47	44.79 ± 5.09	44.82 ± 3.61	>0.05	44.75 ± 2.97	43.17 ± 3.68	43.40 ± 3.22	>0.05
GLB (g/L)	28.51 ± 2.53	27.98 ± 4.02	31.55 ± 2.78	>0.05	28.93 ± 4.17	29.18 ± 5.05	30.79 ± 2.91	>0.05
Cr (μmol/L)	78.53 ± 11.25	75.79 ± 12.47	78.88 ± 13.36	>0.05	80.51 ± 14.41	71.85 ± 10.85	74.49 ± 13.31	>0.05
BUN (mmol/L)	5.27 ± 1.40	4.94 ± 1.06	5.18 ± 1.46	>0.05	5.28 ± 1.53	4.64 ± 1.25	4.97 ± 1.45	>0.05
HBeAg+ (n, %)	19 (54.3%)	9 (37.5%)	N/A	>0.05†	13 (76.5%)	6 (50.0%)	N/A	>0.05†
HBV DNA (log10 copies/mL)	5.82 ± 2.18	5.78 ± 1.74	N/A	>0.05	5.97 ± 1.58	5.27 ± 1.97	N/A	>0.05
PLT (10^9^/L)	208.80 ± 68.19	159.71 ± 61.31	213.22 ± 46.68	**<0.01**	206.53 ± 65.45	159.83 ± 44.99	214.48 ± 30.44	**<0.01**
HDL-C (mmol/L)	1.36 ± 0.26	1.23 ± 0.32	1.26 ± 0.59	>0.05	1.20 ± 0.21	1.23 ± 0.32	1.11 ± 0.39	>0.05
LDL-C (mmol/L)	2.71 ± 0.86	2.73 ± 0.66	2.60 ± 0.70	>0.05	2.53 ± 0.58	2.42 ± 0.60	2.51 ± 0.72	>0.05
CHL (mmol/L)	4.70 ± 0.96	4.62 ± 0.77	4.48 ± 0.93	>0.05	4.19 ± 0.72	4.14 ± 0.79	4.53 ± 0.77	>0.05
TG (mmol/L)	1.19 ± 0.69	1.43 ± 1.08	1.20 ± 0.58	>0.05	1.00 ± 0.27	1.02 ± 0.32	1.92 ± 1.90	>0.05
GLU (mmol/L)	4.79 ± 0.40	5.03 ± 0.48	5.06 ± 0.79	>0.05	5.04 ± 0.26	5.13 ± 0.36	5.10 ± 0.54	>0.05
Liver necroinflammation	G0: 1	G0: 0	NA	**<0.05**#	G0: 0	G0: 0	NA	<0.05#
	G1: 34	G1: 1			G1: 17	G1: 2		
	G2: 0	G2: 14			G2: 0	G2: 8		
	G3: 0	G3: 9			G3: 0	G3: 1		
	G4: 0	G4: 0			G4: 0	G4: 1		
Liver fibrosis	S0: 20	S0: 0	NA	**<0.05**#	S0: 12	S0: 0	NA	<0.05#
	S1: 15	S1: 11			S1: 5	S1: 5		
	S2: 0	S2: 9			S2: 0	S2: 4		
	S3: 0	S3: 2			S3: 0	S3: 1		
	S4: 0	S4: 1			S4: 0	S4: 2		

ALT, alanine aminotransferase; AST, aspartate aminotransferase; ALB, albumin; GLB, globulin; CR, creatinine; BUN, blood urea nitrogen; GGT, γ-glutamyltransferase; AKP, alkaline phosphatase; HbeAg, hepatitis B e antigen; PLT, platelet; HDL-C, high-density lipoprotein cholesterol; LDL-C, low-density lipoprotein cholesterol; TCHL, toal cholesterol; TG, triglyceride; GLU, blood glucose level.

CHB1 and CHB2, patients showing mild and intermediate liver necroinflammation and fibrosis.

^*^OneWay ANOVA or otherwise indicated.

^†^Chi-square test.

^#^Kruskal Wallis test for ordinal variables.

**Table 2:** Discriminating serum metabolites.

RT (min)	MZ (Th)	Identification (shortname)^#^	VIP (PLS-DA)	FC CHB1vsCon	Ttest CHB1vsCon	FC CHB1vsCHB2	Ttest CHB1vsCHB2
7.53	297.1674	3-Hydroxytetradecanedioic acid (3HTDEA)	8.26	0.42	1.15E-09	1.54	>*0.05*
8.09	274.2736	C16 Sphinganine	4.12	1.38	0.034789	1.30	>*0.05*
8.39	325.1977	9-hydroxy-hexadecan-1,16-dioic acid (9HHDDA)	3.46	0.54	0.000274	1.75	>*0.05*
8.46	437.1942	*Unknown compound 1*	10.68	0.87	>*0.05*	1.51	0.0014
9.04	525.3042	*Unknown compound 2*	5.13	0.53	0.000338	1.80	>*0.05*
9.47	553.3356	Lithocholate 3-O-glucuronide (LCA-3-O-GCN)	6.52	0.50	4.56E-05	1.57	>*0.05*
10.08	639.4106	*Unknown compound 3*	7.04	0.54	0.000191	1.39	>*0.05*
10.66	520.3403	LysoPC (18:2)_C_26_H_50_NO_7_P_1* (LysoPC_1)	2.48	1.09	>*0.05*	0.69	0.0004
10.87	566.3216	PC (0:0/20:4)_C_28_H_50_NO_7_P (PC_1)	2.45	0.95	>*0.05*	0.82	0.0056
10.93	520.3408	LysoPC (18:2)_C_26_H_50_NO_7_P_2* (LysoPC_2)	6.06	1.32	3.88E-06	0.85	0.0065
11.14	496.3404	PC (0:0/16:0)_C_24_H_50_NO_7_P (PC_2)	3.63	0.96	>*0.05*	0.85	0.0063
11.35	659.2885	*Unknown compound 4*	4.34	1.48	1E-05	1.14	>*0.05*
11.44	991.6747	N-acetylneuraminyl-Galactosylceramide (GM4)	11.08	0.85	0.016832	0.73	0.0002
11.46	496.3405	PC (16:0/0:0)_C_24_H_50_NO_7_P (PC_3)	7.57	1.16	3.01E-05	0.91	0.0178
11.46	497.3427	2-Palmitoylglycerophosphocholine (2PGPC)	5.47	1.08	>*0.05*	0.68	9E-05
11.74	544.3385	LysoPC (20:4)_C_28_H_50_NO_7_P (LysoPC_3)	3.62	1.12	0.001982	0.91	0.0342
11.88	256.2642	Palmitic amide	7.43	0.19	3.18E-22	3.55	0.0281
12.21	282.2796	Oleamide	9.29	0.24	3.8E-14	4.64	0.0258
12.77	524.3715	LysoPC (18:0)_C_26_H_54_NO_7_P (LysoPC_4)	6.00	1.14	0.00194	0.93	>*0.05*
12.83	805.5167	PI (P-16:0/17:2)_C_42_H_77_O_12_P (PI)	2.90	0.04	2.97E-14	0.02	4E-11
15.25	796.5467	PS (P-18:0/20:4)_C_44_H_78_NO_9_P (PS)	4.04	4.95	5.96E-06	0.59	0.025
17.04	828.5525	PC (22:5/18:4)_C_48_H_78_NO_8_P (PC_4)	3.23	1.73	0.004687	1.85	0.001
17.47	808.5877	PC (20:4/18:1)_C_46_H_82_NO_8_P (PC_5)	3.15	1.40	3.88E-07	1.43	1E-06
17.52	778.0394	*Unknown compound 5*	2.77	0.32	4.57E-06	0.19	1E-10
18.03	760.5857	PC (19:1/15:0)_C_42_H_82_NO_8_P (PC_6)	4.81	1.45	3.78E-11	1.18	0.005
18.27	786.6019	PC (18:1/18:1)_C_44_H_84_NO_8_P (PC_7)	5.92	1.49	1.02E-16	1.17	0.0016

LysoPC, lysophosphatidylcholine; PC, phosphatidylcholine; PI, phosphatidylinositol; PS, phosphatidylserine.

^*^These two PC isoforms share exact the same m/z and chemical formula, but were eluted at different RT.
