# Supplementary material for: Serum metabolomic signatures discriminate early liver inflammation and fibrosis stages in patients with chronic hepatitis B
Source: Sci Rep. 2016 Aug 8;6:30853. doi: 10.1038/srep30853 (PMC4976343; doi:10.1038/srep30853)
Supplement: Supplementary Information [file srep30853-s1.doc]

Serum metabolomic signatures discriminate early liver inflammation and fibrosis stages in patients with chronic hepatitis B

Haijun Huang, Zeyu Sun, Hongying Pan, Meijuan Chen, Yongxi Tong, Jiajie Zhang, Deying Chen, Xiaoling Su, Lanjuan Li

**Supporting Information**

**Patients enrollment**

Eighty-eight consecutive treatment naive CHB patients were prospectively enrolled in Department of infection disease, Zhejiang provincial people’s hospital from June 2012 to December 2013. Inclusion criteria were age ≥ 20 years, positive HBsAg for more than 6 months, HBV DNA ≥ 103 copies/ mL and ALT ≤2 ULN (ULN=50 U/L); ALT and HBV DNA were monitored monthly for 6 months prior to enrollment to ensure the persistent maintenance of ALT ≤2 ULN and HBV DNA ≥ 103 copies/mL. The control group included 67 healthy individuals who came to the hospital for medical evaluation. They were confirmed to have normal liver function without any liver diseases. Exclusion criteria included co-infection with human immunodeficiency virus, other hepatitis viruses, compensated or decompensated liver cirrhosis, alcoholic or non-alcoholic fatty liver diseases, autoimmune liver diseases, hepatocarcinoma, HBV DNA negative, renal insufficiency, and incomplete clinical data.

**Serum sample collection and preparation**

Peripheral venous blood samples were collected and allowed to clot for a minimum of 1 h at 4 °C. The serum fraction is prepared by centrifugation of the blood collection tube at 2,500g for 15 min at 4 °C to remove debris, and the resulting supernatants were aliquoted for analyses or immediately stored at -80 °C.

**Biochemical measurement**

Biochemical tests for alanine aminotranferease (ALT), aspartate aminotransferase (AST), alkaline phosphatase (ALP), gamma-glutamyl transferase (GGT), total bilirubin (Tbil), albumin (ALB) were performed in the clinical laboratory using commercially available assays. Hepatitis antibodies HBsAg, HBsAb HBeAg, HBeAb, HBcAb, and anti-HCV were measured using CLIA-approved (Clinical Laboratory Improvement Act) systems compatible with AASLD practice guidelines, including anti-HCV kit (Xin Chuan Bioteh Co. Ltd., Dalian, Liaoning, China. The HBsAg, HBeAg and antibody to the hepatitis B e antigen (anti-HBe) were measured using commercially available immunoassays (Abbott Laboratories, Chicago, IL). The serum HBV-DNA levels were measured by a quantitative HBV-DNA PCR kit (Da An Gene Co. Ltd., Xiamen, Fujian, China) with a linear detecting range of 300 to 10^8^ copies/mL and detected with a real-time polymerase chain reaction (PCR) system (ABI7300, Applied Biosystems, Foster City, CA, USA).

**Liver biopsy and histopathological analysis**

All enrolled patients received a percutaneous LB using an 18G biopsy needles directed by ultrasonography. The specimens were ﬁxed with formalin and embedded in paraffin. Finally, they were stained with hematoxylin and eosin (HE) and masson’s trichrome for light microscopic examination. A minimum of 1.5 cm of liver tissue with at least six portal tracts was required for diagnosis. Two experienced clinical pathologists examined the sections for grading of liver necroinflammation activity (G0-G4) and staging of liver fibrosis (S0-S4) using Scheuer’s classiﬁcation [^1^](#_ENREF_1). Liver necroinflammation activity was considered significant when G≥2. Liver fibrosis was considered significant when it spread beyond the portal tract (S≥2).

**Metabolomic profiling**

Serum metabolite fingerprinting was performed on a UPLC-HRMS system consisted of a Waters ACQUITY UPLC system hyphened with a Waters Q-TOF Premier mass spectrometer (both from Waters Co., Milford, MA). Samples were diluted 1:1 with HPLC water and were randomly assigned to a test queue before injection (10 μL) into a reversed-phase column (ACQUITY 100×2.1mm, 1.7μm, Waters, Milford, MA, USA) at 50 °C. The system was operated at a flow rate of 0.3 mL/min of mobile phase consisting of solvent A: water with 0.1% formic acid (FA, Sigma-Aldrich, St. Louis, MO, USA), and solvent B: acetonitrile (ACN, Sigma-Aldrich) with 0.1% FA. The total analysis time per sample was 25 min. The gradient started with 3% of B and increased to 80% in 7.5 min, then reached 98% in 8 min and maintained for 5 min. The gradient finally reached 100% B in 0.5 min and was held for 3 min before it return to 3% B in 1 min and kept 8 min re-equilibration until next injection. All MS analyses were performed on ESI+ mode with full MS scan using parameters detailed in the supplementary data. A quality control (QC) sample was pooled equally from all samples, and was inserted between every 8 runs throughout the experiment.

**UPLC-QToF data processing**

The raw data were recorded by MassLynx v4.1 (Waters Co., Milford, MA) in centroid mode. Subsequently, background noise subtraction, peak detection, and peak area normalization against the total ion current of each sample were performed using MZmine 2 software[^2^](#_ENREF_2). Individual compounds were recognized by unique m/z and retention time values (hereafter RT-m/z features). Chromatograms of each RT-m/z feature were then aligned across samples/injections for comparison. Metabolite features were then tabulated with unique m/z, RT, and normalized peak areas from each sample and exported for downstream multivariate analyses. Using the minimum intensity threshold of 200 counts, a total of 4636 unique RT-m/z features were consistently detected in at least 60% serum samples, and were subsequently used for multivariate analyses.

**Multivariate Modeling and Statistical Analyses**

To build sound multivariate models, only RT-m/z features presented in 60% of all samples across the whole experiment were chosen. Data were mean centered and pareto scaled before Partial Least Squares Projection to Latent Structures regression with Discriminant Analysis (PLS-DA) to model intergroup differences. To guard against model over-fitting, all PLS-DA models were subjected to 7-fold cross-validation with 500 random permutations. Further simplified orthogonal PLS-DA models based on short lists of markers that differentiate CHB2 from CHB1 and CHB1 from controls were built via the similar approach. All PLS-DA and OPLS-DA models were developed by SIMCA-P v14.1 (Umetrics AB, Sweden).

All other statistical analyses for clinical variables were performed within the R environment. Quantitative variables are expressed in mean +-s.d. and qualitative variables in absolute and relative frequencies. Continuous data were compared either by one-way ANOVA or student's *t*-test, while categorical data were compared using Chi-square test. Significance was established by *P*<0.05. ROC analyses were performed by ROCR pack in R. The sensitivity and specificity of each marker was calculated at the best cut-off value.

**Biomarker Selection and Metabolite Identification**

By convention, variable with VIP>1 (Variable Importance in the Project score) can be considered as potential contributor to the PLS-DA model. Here we take extra stringent criteria of VIP>2 based on PLS-DA model, plus intergroup student's *t*-test (*P* < 0.05) for each individual RT-m/z feature to select potential biomarkers candidate for CHB staging. To avoid spurious identification commonly see in low mass range in LC-MS data due to background or solvent contaminants, only species with m/z > 100 were selected. To further pave the path for future robust assay development, species with average MS intensity<1000 counts, LC retention time RSD>0.5% were discarded.

Collision induced dissociation (CID) assisted MS/MS spectra of each metabolite candidate were collected for structure elucidation of potential biomarker candidates. UPLC and mass spectrometry parameters for MS/MS experiment were exactly the same as previously described except the quadruple was set to isolate candidate precursor m/z during the whole UPLC gradient, while the TOF mass analyzer operated in full scan mode from m/z 50 to 1000 Th. Argon was used as the collision gas with different collision energies ranging from 30 to 80 V, according to the respective chemical stability. The UPLC-MSMS data were collected by MassLynx v4.1 software (Waters Co., Milford, MA). Tandem spectra of each metabolite candidate were exported in mgf format. To identify potential biomarkers, both HMDB (http://www.hmdb.ca/), METLIN (http://metlin.scripps.edu/) were searched using MS peak list with 5 and 10 ppm mass error for precursor and product ions, respectively. Compounds without MS/MS spectra documented in HMDB or METLIN were identified with accurate precursor match at < 5 ppm mass tolerance. For multiple ambiguous matches with identical molecular weight and chemical formula, compound classes at the lowest granular level of chemical taxonomy were reported.

**Pathway Analysis**

For pathway analysis, a total of 455 significantly changed RT-m/z features (student’s *t*-test, *P* <0.001) from CHB2 vs. CHB1 comparison were matched to potential metabolite candidates at m/z tolerance of 10 ppm using mummichog (v.1.0.5) approach. The matched candidates were then mapped to reference human metabolic networks from the KEGG , MetaCyc [^5^](#_ENREF_5), Recon and Edinburgh Human Metabolic Network [^8^](#_ENREF_8). Enrichment significance of each pathway was computed by Fisher exact test using all RT-m/z features as reference background. The null distribution in pathway analysis was obtained from 1000 set of randomly permutated m/z lists draw from all 4636 m/z features detected in the whole metabolomic dataset and modeled by Gamma distribution. The premise of this metabolic network prediction strategy is founded on the assumption that metabolite concentration alterations are more likely to occur within a metabolic connected network rather than in a random fashion. Therefore, even without prior knowledge of the *bona fide* metabolites, one can exhaustively list all possible metabolite compounds from a list m/z and to test if such connected networks can be enriched. This high throughput approach has been validated on multiple recent metabolomic studies [^9-12^](#_ENREF_9).

**Suppl Table 1.** Summary of multivariate models constructed

|  | Cross validation | | | Class permutation test | |
| --- | --- | --- | --- | --- | --- |
| model | R^2^X | R^2^Y | Q^2^Y | R intercept | Q intercept |
| PLS-DA w/ full list | 0.45 | 0.78 | 0.62 | 0.38 | -0.30 |
| OPLS-DA w/ 21 markers  CHB1vsCon | 0.48 | 0.65 | 0.58 | 0.10 | -0.23 |
| OPLS-DA w/ 18 markers  CHB2vsCHB1 | 0.42 | 0.67 | 0.57 | 0.14 | -0.31 |

**Suppl Table 2.** Comparison of diagnostic characteristics of clinical indicators and potential metabolite markers combinations

|  | ***AUC*** | ***SE*** | ***Cut-off*** | ***95% CI*** | ***Sensitivity (%)*** | ***Specificity (%)*** |
| --- | --- | --- | --- | --- | --- | --- |
| ***Individual Markers*** | | | | | | |
| ALT | 0.709 | 0.0600 | >34 | 0.591 to 0.827 | 72.22 | 71.15 |
| AST | 0.765 | 0.0516 | > 31 | 0.664 to 0.866 | 69.44 | 76.92 |
| ALB | 0.581 | 0.0632 | ≤46.9 | 0.457 to 0.705 | 72.22 | 46.15 |
| AKP | 0.728 | 0.0569 | >76 | 0.616 to 0.839 | 72.22 | 67.31 |
| GGT | 0.701 | 0.0578 | >19 | 0.588 to 0.814 | 72.22 | 57.69 |
| PLT | 0.718 | 0.0550 | ≤202 | 0.610 to 0.826 | 86.11 | 48.08 |
| BUN | 0.587 | 0.0613 | ≤5.45 | 0.467 to 0.707 | 77.78 | 44.23 |
| Cr | 0.613 | 0.0620 | ≤68.5 | 0.491 to 0.735 | 44.44 | 78.85 |
| GLB | 0.525 | 0.0672 | ≤24.3 | 0.393 to 0.657 | 22.22 | 94.23 |
| GM4 | 0.743 | 0.0526 | > 275.901 | 0.640 to 0.846 | 80.56 | 65.38 |
| LysoPC_1 | 0.745 | 0.0543 | > 16.0956 | 0.638 to 0.851 | 77.78 | 71.15 |
| PC_5 | 0.770 | 0.0532 | ≤14.9486 | 0.665 to 0.874 | 52.78 | 94.23 |
| PI | 0.870 | 0.0417 | >4.7931 | 0.789 to 0.952 | 75.00 | 100 |
| PS | 0.707 | 0.0577 | >5.2162 | 0.594 to 0.820 | 100 | 44.23 |
| **PIPSindex** | **0.961** | 0.0233 | >0 | 0.916 to 1 | 83.33 | 100 |

ALT, alanine aminotransferase; AST, aspartate aminotransferase; ALB, albumin; GLB, globulin; Cr, creatinine; BUN, blood urea nitrogen; GGT, γ-glutamyltransferase; AKP, alkaline phosphatase; PLT, platelet. GM4, N-acetylneuraminyl-galactosylceramide; LysoPC, lysophosphatidylcholine; PC, phosphatidylcholine; PI, phosphatidylinositol; PS, phosphatidylserine.

CHB1 and CHB2, patients showing mild and intermediate liver necroinflammation and fibrosis.

**Suppl Table 3.** Pathway Analysis

| **Pathways** | **Overlap size** | **Pathway size** | **p-value** |
| --- | --- | --- | --- |
| De novo fatty acid biosynthesis | 6 | 13 | 0.00069 |
| Vitamin E metabolism | 9 | 27 | 0.00072 |
| Fatty acid activation | 5 | 13 | 0.00117 |
| Vitamin A (retinol) metabolism | 5 | 17 | 0.00258 |
| Porphyrin metabolism | 4 | 13 | 0.00371 |
| Methionine and cysteine metabolism | 5 | 19 | 0.00403 |
| Androgen and estrogen biosynthesis and metabolism | 5 | 20 | 0.00506 |
| Polyunsaturated fatty acid biosynthesis | 2 | 3 | 0.00539 |
| Omega-3 fatty acid metabolism | 2 | 4 | 0.00945 |
| Urea cycle/amino group metabolism | 4 | 18 | 0.01392 |
| Glycine, serine, alanine and threonine metabolism | 3 | 13 | 0.02208 |
| Carnitine shuttle | 4 | 20 | 0.02237 |
| Squalene and cholesterol biosynthesis | 4 | 20 | 0.02237 |
| Glycerophospholipid metabolism | 4 | 21 | 0.02792 |
| Bile acid biosynthesis | 5 | 29 | 0.03373 |
| Leukotriene metabolism | 6 | 36 | 0.03379 |

Enrichment analysis was performed by mummichog algorithm based on 455 significantly changed m/z species.

| a  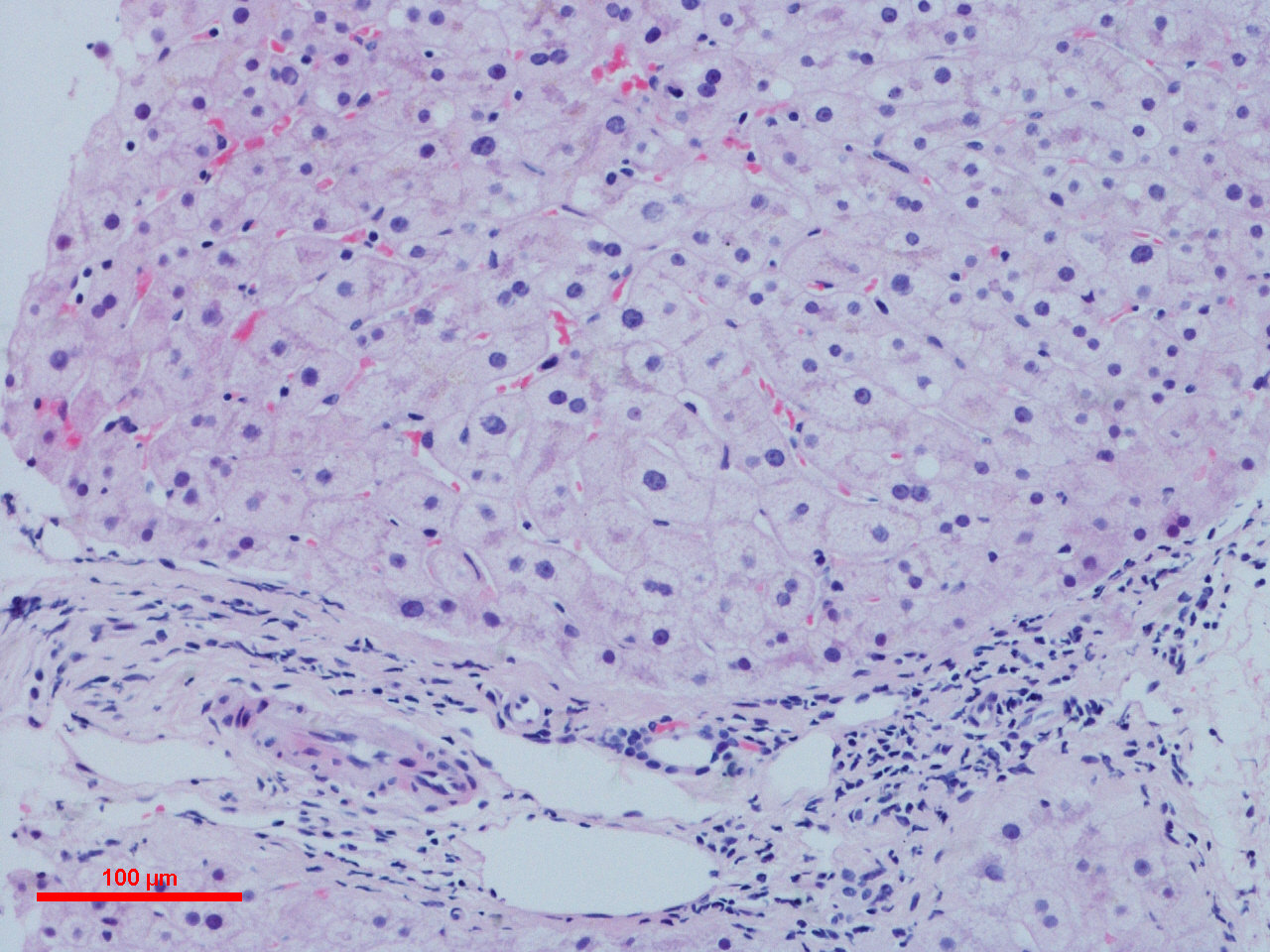 | b  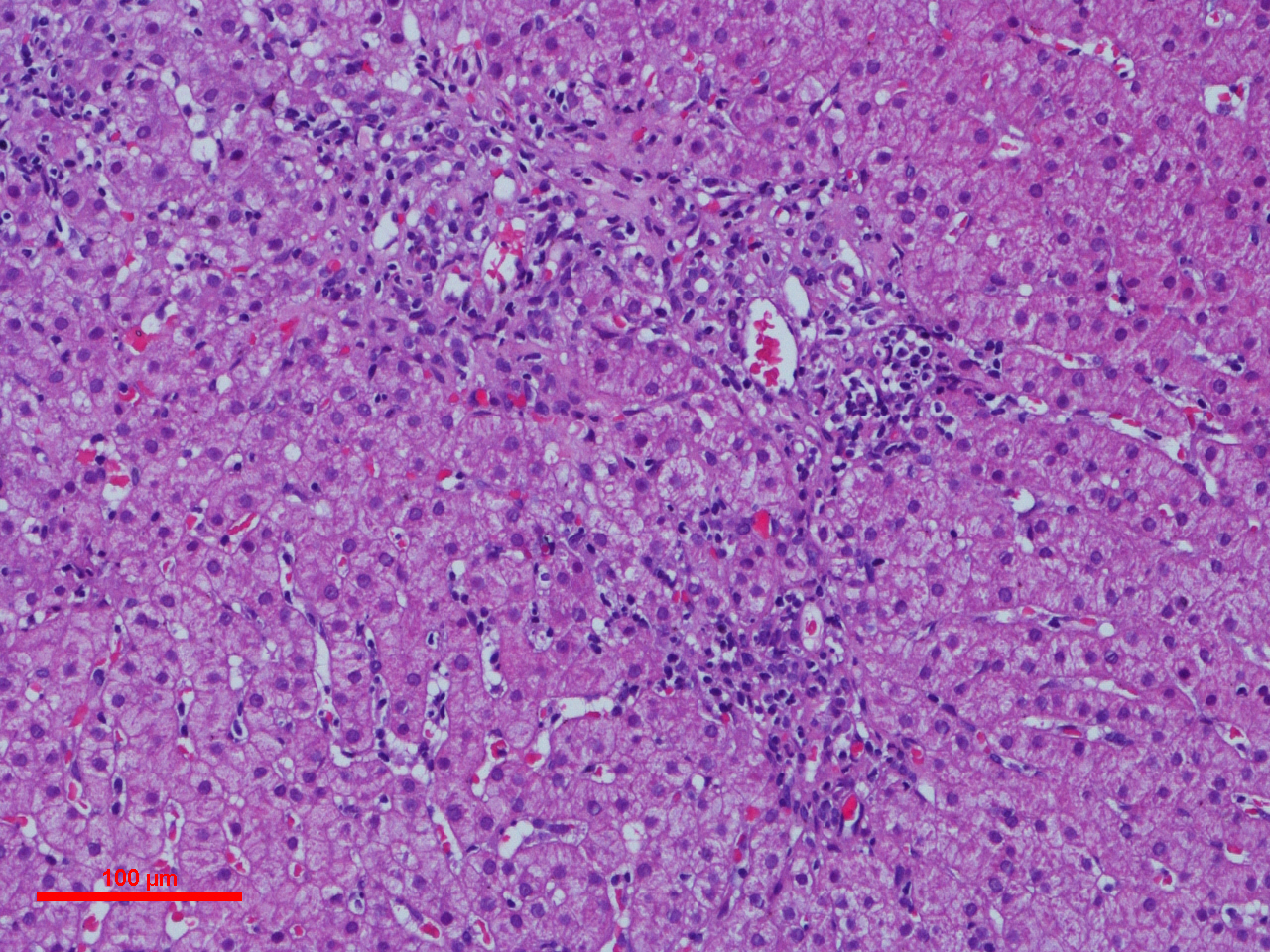 |
| --- | --- |
| Supplementary Fig. 1. Liver biopsy histopathology (× 200) from patients with necroinflammation at G1S1 (a) and G3S2 (b) stages. Biopsy samples were stained by hematoxylin and eosin method. More infiltrated lymphocytes around lobules were found in the G3S2 biopsy than that in the G1S1 biopsy. Significant hepatocytes necrosis, cytoplasma swelling can be found in the G3S2 biopsy. | |

| a  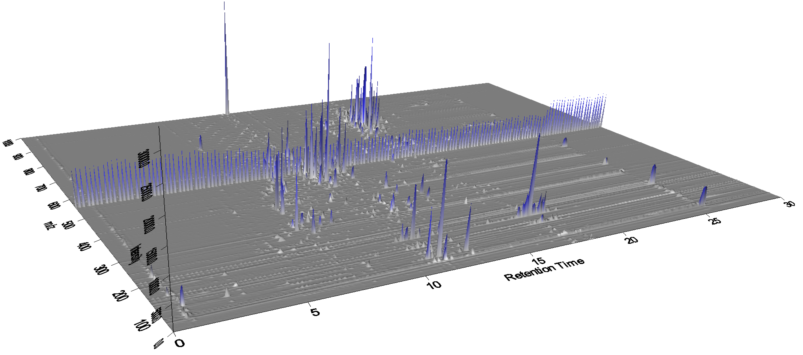 | b |
| --- | --- |
| Supplementary Fig. 2. Typical chromatogram of UPLC-HRMS data in 3D format (a), features were identified by their unique combination of retention time and m/z measurement. Peak intensity was used to compare the relative abundance of each feature across multiple samples. Instrument performance was monitored by QC samples injected between every 8-9 samples across the whole experiment, chromatogram alignment of all QC samples were also shown (b). | |

| a  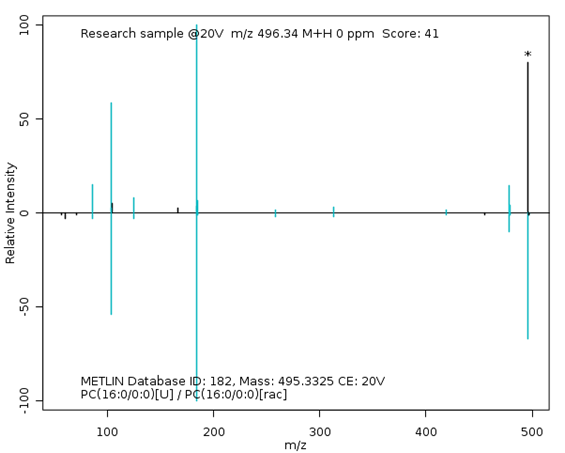 | b  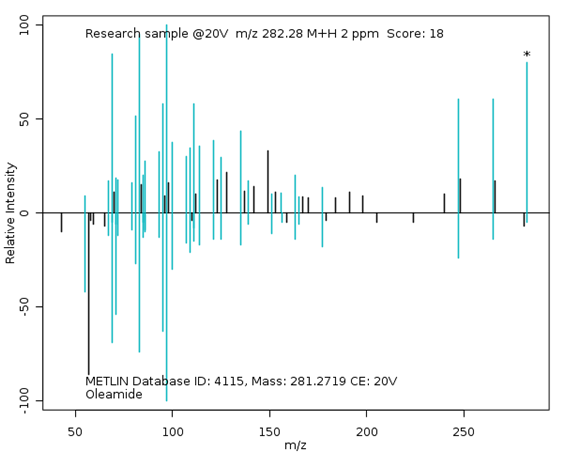 |
| --- | --- |
| Supplementary Fig. 3, Biomarker identification by matching experimental CID MS2 spectra compared to candidate spectra from Metlin database. The matched fragment peaks are in blue while unmatched ones in black. * refer to the precursor. | |

| 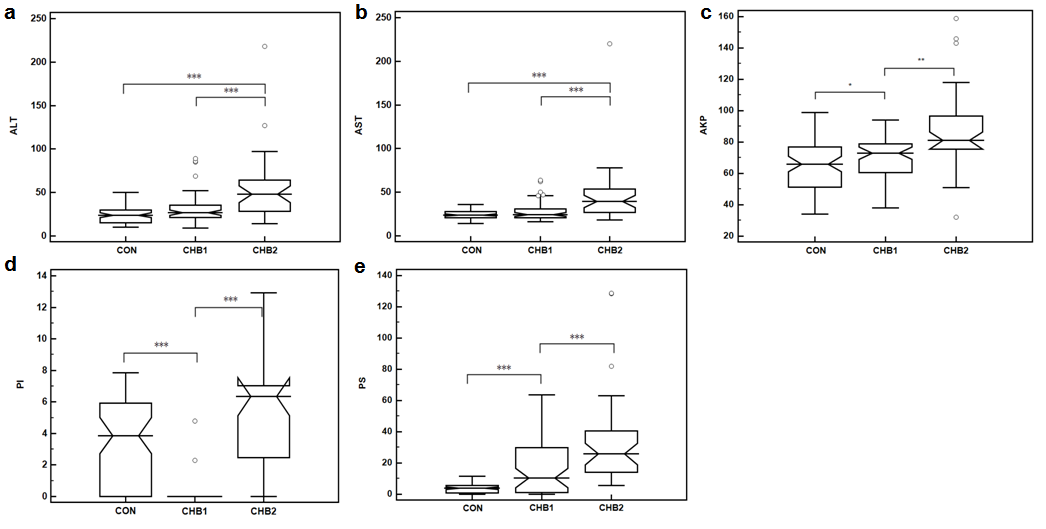 |
| --- |
| Supplementary Fig. 4, Relative abundance of biomarkers in control, CHB1 and CHB2 patients. Notched boxes denote interquartile ranges, lines denote medians and whiskers denote 10th and 90th percentiles. ALT, AST and AKP level (a-c) is recorded as IU/L. The relative abundance of PI and PS (d,e) refers to peak area in 1/10000th the total ion current (TIC). Statistical p value was calculated using a Student's t-test. *p<0.05, **p<0.01, ***p<0.001.  PI, phosphatidylinositol; PS, phosphatidylserine |

Reference for the supplementary materials

1 European Association For The Study Of The, L. EASL Clinical Practice Guidelines: management of chronic hepatitis B. *J Hepatol.* **50**, 227-242 (2009).

2 Pluskal, T., Castillo, S., Villar-Briones, A. & Oresic, M. MZmine 2: modular framework for processing, visualizing, and analyzing mass spectrometry-based molecular profile data. *BMC Bioinformatics.* **11**, 395 (2010).

3 Kanehisa, M., Goto, S., Sato, Y., Furumichi, M. & Tanabe, M. KEGG for integration and interpretation of large-scale molecular data sets. *Nucleic Acids Res.* **40**, D109-114 (2012).

4 Tanabe, M. & Kanehisa, M. Using the KEGG database resource. *Curr Protoc Bioinformatics.* **Chapter 1**, Unit1 12 (2012).

5 Caspi, R. *et al.* The MetaCyc database of metabolic pathways and enzymes and the BioCyc collection of pathway/genome databases. *Nucleic Acids Res.* **40**, D742-753 (2012).

6 Mo, M. L., Jamshidi, N. & Palsson, B. O. A genome-scale, constraint-based approach to systems biology of human metabolism. *Mol Biosyst.* **3**, 598-603 (2007).

7 Thiele, I. *et al.* A community-driven global reconstruction of human metabolism. *Nat Biotechnol.* **31**, 419-425 (2013).

8 Ma, H. *et al.* The Edinburgh human metabolic network reconstruction and its functional analysis. *Mol Syst Biol.* **3**, 135 (2007).

9 Hariharan, R. *et al.* Invariance and plasticity in the Drosophila melanogaster metabolomic network in response to temperature. *BMC Syst Biol.* **8**, 139 (2014).

10 Hoffman, J. M. *et al.* Effects of age, sex, and genotype on high-sensitivity metabolomic profiles in the fruit fly, Drosophila melanogaster. *Aging Cell.* **13**, 596-604 (2014).

11 Li, S. *et al.* Predicting network activity from high throughput metabolomics. *PLoS Comput Biol.* **9**, e1003123 (2013).

12 Xu, X. *et al.* Autophagy is essential for effector CD8(+) T cell survival and memory formation. *Nat Immunol.* **15**, 1152-1161 (2014).
